# Epigenetic Mechanisms Underlying Adult Post Stroke Neurogenesis

**DOI:** 10.3390/ijms21176179

**Published:** 2020-08-27

**Authors:** Xianshuang Liu, Baoyan Fan, Michael Chopp, Zhenggang Zhang

**Affiliations:** 1 Department of Neurology, Henry Ford Health System, Detroit, MI 48202, USA; bfan1@hfhs.org (B.F.); mchopp1@hfhs.org (M.C.); zzhang1@hfhs.org (Z.Z.); 2 Department of Physics, Oakland University, Rochester, MI 48309, USA

**Keywords:** stroke, epigenetics, microRNAs, histone deacetylation, adult neurogenesis, long non-coding RNA

## Abstract

Stroke remains the leading cause of adult disability. Post-stroke neurogenesis contributes to functional recovery. As an intrinsic neurorestorative process, it is important to elucidate the molecular mechanism underlying stroke-induced neurogenesis and to develop therapies designed specifically to augment neurogenesis. Epigenetic mechanisms include DNA methylation, histone modification and its mediation by microRNAs and long-non-coding RNAs. In this review, we highlight how epigenetic factors including DNA methylation, histone modification, microRNAs and long-non-coding RNAs mediate stroke-induced neurogenesis including neural stem cell self-renewal and cell fate determination. We also summarize therapies targeting these mechanisms in the treatment of stroke.

## 1. Introduction

Stroke is one of the leading causes of morbidity and mortality in the U.S. and worldwide, which often leads to long-term neurological deficits and disability. Treatments of ischemic stroke with thrombolysis using tissue plasminogen activator (tPA) for the treatment of acute stroke patients within 4.5 h and mechanical endovascular thrombectomy for patients with acute ischemic stroke within 24 h due to a large artery occlusion results in only approximately one third of patients experiencing early brain reperfusion [1]. However, approximately two thirds of patients remain with neurologic impairment and disability. Therefore, post stroke rehabilitation becomes a major therapeutic focus for most stroke patients. Unfortunately, the currently available therapies are only rarely successful in improving recovery from neurological deficits. Neural stem cells are present in the adult brain of human and rodent [2,3,4,5,6,7,8,9,10,11]. Neurogenesis contributes to improvement of neurological function [3,7,8,9,10,11,12,13,14,15,16,17,18,19,20,21,22,23]. Although stroke induces stem cells to generate new neurons, post-stroke neurogenesis is modest and insufficient to restore neurological function [8,9,10,11,23]. It is therefore vital to identify the molecular and cellular signals required to promote neurogenesis after stroke.

Epigenetics is the study of heritable changes in gene function that cannot be attributed to DNA sequence variations. Epigenetic mechanisms can regulate different physiological processes that occur in a living organism, including cellular proliferation and differentiation. A deeper knowledge of the epigenetic modifications related to stroke and its risk factors could provide the basis for the development of innovative approaches in the prevention and treatment of this disabling disease. In the last decade, evidence has emerged supporting the involvement of epigenetic mechanisms in many neurodegenerative diseases and stroke [24,25,26]. In this review article, we describe the different types of epigenetic mechanisms and their involvement in the pathogenesis of stroke-induced neurogenesis.

## 2. Stroke-Induced Neurogenesis

In the adult rodent brain, neurogenesis occurs throughout life in two discrete regions, primarily in the subventricular zone (SVZ) of the lateral ventricle and in the subgranular zone (SGZ) of the dentate gyrus (DG) [3,27,28,29]. Neurogenesis after stroke is present in peri-infarct regions and is essential for brain repair processes and cognitive restoration during stroke recovery [30,31,32]. We and others demonstrated that focal cerebral ischemia dramatically increases proliferation of SVZ progenitor cells in rodents and human [3,13,14,17,23,33]. Following proliferation, many SVZ neuroblasts abandon the rostral migratory system/olfactory complex [34] and migrate laterally toward the ischemic boundary zone [8,9,10,11,13,33,35,36,37,38]. Upon arrival, some SVZ neuroblasts integrate and differentiate, expressing markers of spiny neo-striatal projection neurons [3,21,23]. Increased neurogenesis in the dentate gyrus following stroke is also associated with impairment of hippocampus-dependent memory, although the extent to which stroke-induced neurogenesis contributes to the hippocampal cognitive function is controversial [39,40,41,42,43]. Ablation of newly generated neuroblasts in ischemic brain reduces spontaneous functional recovery and causes cognitive deficits in a mouse model of middle cerebral artery occlusion (MCAO) [20,42,43,44], indicating that stroke-induced neurogenesis impacts neurological outcomes. In addition, neurogenesis contributes to axonal remodeling, dendrite outgrowth [21,45] and synapse connectivity [21,45]. It has also been shown that even re-establishment of a fraction of damaged neuronal circuits substantially improves neurological function [33]. Stroke-induced neurogenesis has also been demonstrated in the adult human brain, even in advanced age patients [7,14,46,47,48]. However, neurogenesis in response to stroke is insufficient to restore neurological function [33,49]. Elucidating molecular mechanisms underlying stroke-induced neurogenesis could lead to the development of new therapies to amplify endogenous neurogenesis, consequently leading to the improvement of neurological function during stroke recovery.

## 3. Epigenetics in Adult Neurogenesis after Stroke

### 3.1. Overview of Epigenetics in the Gene Regulation

Epigenetics is critical for normal development and cell growth and likely provides an important interface among genes, environment and disease. Epigenetic abnormalities have been found to be causative factors in numerous diseases [50]. Epigenetic mechanisms include histone covalent post-translational modifications, introduction of histone variants, chromatin remodeling, DNA methylation, imprinting and RNA interference [50]. We highlight these major epigenetic mechanisms in different stages of adult neurogenesis in the SVZ and SGZ/dentate gyrus after stroke.

### 3.2. DNA Methylation and Demethylation

DNA methylation involves the chemical covalent modification of the 5-carbon position of cytosine to produce 5-methylcytosine (5mC) on CpG islands, which renders the chromatin more tightly closed and inaccessible for the transcriptional machinery leading to silencing of gene expression. A family of DNA methyltransferases (Dnmts) is responsible for the catalysis of DNA methylation for preserving or generating 5mCs on the genome. In addition, methyl-CpG-binding domain (MBD) family members, such as MBD1 and MeCP2, can also bind methylated CpGs in the promoters and suppress gene expression [51]. Recent studies have revealed that knockdown of Dnmt1, Dnmt3a, MBD1 and MeCP2 significantly influence both proliferating NPCs and differentiated neurons by its direct epigenetic regulation of many neurogenic genes [52,53]. Moreover, previous research investigating epigenetic changes following MCAO in mice demonstrated upregulated global DNA methylation within the brain, and genetic or pharmacologic DNMT inhibition using 5-aza-dC, a MTase inhibitor decreased stroke severity [54,55], suggesting that DNA methyltransferase contributes to delayed ischemic brain injury.

In contrast with DNA methylation, a DNA demethylation mechanism has been recently discovered, which involves the removal of the methyl group by actively reversing the form 5mC to unmodified cytosine (C) through the ten-eleven translocation (TET) proteins-mediated oxidation of 5mC to 5-hydroxymethylcytosine (5hmC), 5-formylcytosine (5fC) and 5-carboxylcytosine (5caC), leading to transcriptional activation and gene expression [56]. Deletion of TET proteins markedly reduced the proliferation of adult NPCs by the increase of gene hypermethylation involved in adult NPC proliferation, for example galanin (Gal), chondroitin sulfate proteoglycan 4 (Ng2) and neuroglobin (Ngb), and reduced their expression in NPCs [57,58,59,60]. These data suggest that DNA methylation and demethylation precisely coordinate neurogenesis. However, understanding the role of dynamic DNA methylation on neurogenesis is still in its infancy. Whether methylation of gene expression plays an important role in the regulation of neurogenesis after stroke has not been investigated. Genome-wide maps of different cytosine modifications at single-base solution during the neural lineage commitment, from ESCs to NSCs to neurons, are clearly needed for future studies. In addition, given the complexity and heterogeneity of neurogenesis, the approaches of epigenomic and gene expression analyses at the single-cell level must be developed as well.

### 3.3. Histone Acetylation and Deacetylation

DNA associates with histone proteins in subunits called nucleosomes that form chromatin. Histones can undergo a number of acetylation and deacetylation modifications catalyzed by histone acetyltransferases (HATs) and histone deacetylases (HDACs), respectively, that can allow or prevent transcription in response to the cellular environment. HDACs are divided into four classes based on their homology and structure. Classes I, II and IV comprise Zn2^+^-dependent HDACs, whereas Class III is made up of the NAD-dependent sirtuins. Numerous synthetic or natural product HDAC inhibitors (HDACi) have been developed for cancers and other neurodegenerative diseases [61,62]. Ischemia alters the expression of multiple HDAC proteins, and these have become popular targets for preclinical neuroprotection studies in stroke [63]. Recent studies have demonstrated protective effects of HDAC inhibition by anti-inflammation and inhibition of proapoptotic factors in acute treatment of focal cerebral ischemia model of rats (1–6 h after stroke) with various compounds including valproic acid, trichostatin A and sodium butyrate [64,65,66,67,68,69,70,71,72,73].

There is a significant body of evidence implying the proneurogenic actions of HATs and HDACs in regulating adult neurogenesis under physiological conditions [74]. For example, treatment with an HDAC inhibitor promoted neurite growth and neurogenesis in primary cortical cultures. The function of HDAC inhibitors during the late stage of stroke remains little known. Our previous study demonstrated that administration of Valproate acid (VPA) at a dose of 100 mg/kg starting 24 h after stroke increased acetylated histone H4 expression in neuroblasts and the number of new neurons in striatal ischemic boundary region, as well as increased oligodendrocytes, myelinated axons and neurogenesis in the peri-infarct region 28 days after stroke, which, in concert, were associated with improved neurological outcome [75]. In a rat model of MCAO, post-insult treatment with sodium butyrate (SB) or trichostatin A (TSA) robustly increased cell proliferation in the SVZ and DG of the ischemic brain, and SB also induced the migration of neural precursors to areas of injury by the regulation of BDNF-TrkB-dependent neurogenesis after ischemic injury [64,76]. These data suggest that modulation of histone acetylation in NPCs is involved in stroke-induced neurogenesis.

Despite the wide usage of HDAC inhibitors, however, the contribution of the individual HDACs to neurogenesis and other neurorestorative mechanisms after stroke remains largely unknown. The action of HDAC enzymes is complex, with some subtypes exhibiting protective effects while others promoting cell death. Furthermore, certain classes of HDAC enzymes are expressed outside of the nucleus where they influence the function of diverse proteins in a non-epigenetic manner. Further exploration of the roles of individual HDACs in regulating adult neurogenesis using conditional knockout mice of different HATs and HDACs will help to refine our understanding of the complex role of these proteins in regulating specific aspects of adult neurogenesis and their potential molecular mechanisms in contributing to stroke pathogenesis and subsequent recovery.

### 3.4. Polycomb Repressive Complex 2 (PRC2) Core Proteins

The core of Polycomb Group protein 2 (PRC2) comprises Ezh2, Suz12, Eed and Jarid2 subunits, which inhibit gene expression by catalyzing histone modifications such as di- and trimethylation on histone H3 (H3K27me2 and H3K27me3) at the promoters of specific genes [77]. The function of PRC2 during development and adult neurogenesis has been studied. Yusuke et al. found that, during neocortical development, Ezh2 and Eed suppress the cortical neurogenesis and promote astrogenesis by repressing the promoter of the proneural gene neurogenin1 [78]. In adult neurogenesis, Zhang et al. reported that conditional knockout of *Ezh2* in NSCs/progenitor cells reduces proliferation, leading to a decrease in the number of neurons in hippocampus and ultimately impairments in spatial learning and memory, contextual fear memory and pattern separation [79]. Our new data show that stroke significantly increased SUZ12 and EZH2 in NSCs derived from SVZ and SGZ, whereas histone active marker H3K4me3 exhibited a significant decrease. Using ChIRP-Western blot and RNA immnunoprecipitation (RIP) assay, chromatin proteins including SUZ12, EZH2 and H3K27me3 were observed to be physically associated with long non-coding RNA (lncRNA) H19, whose association was significantly higher in the ischemic NSCs compared with those from non-ischemic NSCs. Furthermore, our data demonstrate that knockdown of lncRNA markedly reduced levels of EZH2 and H3K27me3 at the promoters of cell cycle-related genes, leading to the upregulation of p27 expression and consequently inhibition of NSC proliferation [80]. Our novel preliminary data suggest that stroke alters histone factors and thereby the recruitment of epigenetic states to target genes, which underlie stroke-induced neurogenesis.

### 3.5. MiRNA and Stroke-Induced Neurogenesis

MicroRNAs are 22-nucleotide noncoding RNAs that can downregulate gene products by translational repression when partially complementary sequences are present in the 3′-untranslated regions (3′-UTR) of the target mRNAs or by directing mRNA degradation. MiRNAs are expressed in a tissue-specific manner and are considered to play important roles in cell proliferation, apoptosis and differentiation [81].

Stroke alters the profiles of miRNAs in the brain and in blood samples obtained from humans and rodents [82,83,84,85]. NSCs express miRNAs and the functions of miRNAs in neurogenesis after stroke have been recently revealed [86,87,88,89,90,91,92,93,94,95] (Table 1). In a global screen study of mature miRNAs, we identified that miR-124a, a neuron-specific miRNA, was dramatically decreased in ischemic SVZ NPCs extracted from a rat MCAO model. Furthermore, we found the Jagged-1 and associated Notch signaling, well-known regulators of neurogenesis, are potential targets of miR-124 in NSCs [96]. In addition, we found that components of the miR-17-92 cluster were significantly upregulated in ischemic NPCs. Overexpression of the miR-17-92 cluster increases the proliferation and survival of NPCs by reducing target PTEN (phosphatase and tensin homolog), while Sonic hedgehog (Shh) pathway recruits N-MYC to regulate miR-17-92 cluster expression in NPCs [97]. Using a transgenic mouse line with conditional ablation of the miR-17-92 cluster in nestin lineage NSCs, ablation of the miR-17-92 cluster significantly reduced the number of proliferating NSCs and neuroblasts and neuronal differentiation in the dentate gyrus (DG) of the hippocampus and significantly impaired hippocampal-dependent learning and memory [98], suggesting a functional role of the miR-17-92 cluster in mediating stroke-induced neurogenesis and functional recovery by the SHH/MYC signaling pathway.

Moreover, using a more comprehensive Ago2-based RNA immunoprecipitation to immunoprecipate Ago2-RNA complexes followed by RNA sequencing (Ago2 RIP-seq) approach, we profiled the miRNomes in NPCs from ischemic rats and found that stroke substantially changed Ago2-associated miRNA profiles in NPCs compared to those in non-ischemic NPCs. We also discovered a new complex repertoire of isomiRs and multiple miRNA–miRNA* pairs and numerous novel miRNAs in the non-ischemic and ischemic NPCs [111]. miR-146a, which was known to regulate immune response, was the most upregulated miRNA in ischemic NPCs. However, the function of miR-146a in the NSCs has not been reported. Our data show that overexpression of miR-146a in NPCs significantly increased their differentiation into O4^+^ OPCs, which was further confirmed in primary OPCs, showing gain-of-function of miR-146a in primary OPCs increased expression of myelin proteins, whereas attenuation of endogenous miR-146a suppressed generation of myelin proteins by targeting IRAK1 expression. Attenuation of IRAK1 in OPCs substantially increased myelin proteins and decreased OPC apoptosis [99].

In addition, other groups identified several other miRNAs that can impact adult neurogenesis after stroke. For instance, miR-210, a known hypoxia-related miRNA, was found to promote the neurogenesis and long-term outcomes by targeting brain derived neurotrophic factor (BDNF) after focal ischemia in mice [103]. Intriguingly, another study revealed that miR-210 influences proliferation and Doublecortin (DCX) positive neuroblast apoptosis by glycolytic activity and increases mitochondrial potential via targeting cytochrome *c* assembly protein (COX10) and iron–sulfur cluster scaffold homolog (ISCU) in mice subjected to MCAO [112]. Inhibition of miR-148b enhances proliferation and differentiation of NSCs via Wnt/β-Catenin Signaling in an ischemic mouse stroke model, which contributes to functional recovery after stroke [107]. Knockdown of miR-365 enhances PAX6-mediated neurogenesis from astrocytes and attenuates neuronal injury in the brain after ischemic stroke [106]. AAV-mediated overexpression of miR-140-5p decreased the levels of IL1rap, IL1rapl1, VEGF and MEGF10 in the ischemic mouse hippocampus and inhibited neurogenesis, indicating a novel early warning biomarker for late-onset post-stroke depression [109,113]. Injection of miRNA-27b inhibitors enhances neurogenesis of SVZ and hippocampus, behavioral function recovery and spatial memory via AMPK activation in a mouse ischemic stroke model [110]. MiRNA-126 promoted neurogenesis in ischemic mouse brain by directly inhibiting its target PTPN9 and activating AKT and ERK signaling pathways, and it further improved neurobehavioral outcomes [114]. Collectively, these data suggest that miRNAs play critical roles in stroke-induced neurogenesis and neurological outcomes post stroke.

### 3.6. Long Non-Coding RNAs and Neurogenesis

Long non-coding RNAs (lncRNAs), non-protein coding transcripts longer than 200 nucleotides, have been suggested to play important roles in the regulation of tissue homeostasis and pathophysiological conditions [115]. LncRNAs regulate gene expression at the levels of chromatin structure, transcription control, RNA processing and translation [115]. Many aberrant lncRNA expressions such as MEG3, H19, MALAT1, ZFAS1, GAS5, LincRNA-EPS, SHNG16, etc. were suggested to regulate cell apoptosis, angiogenesis, inflammation and cell death in different brain cell types after stroke [116,117,118,119,120,121,122]. NSCs express lncRNAs and the functions of lncRNAs in the self-renewal and differentiation of stem cells are beginning to be appreciated [123,124,125]. In our recent study, we performed a comprehensive lncRNA profile analysis from adult NSCs in the SVZ of adult non-ischemic and ischemic rats subjected to seven-day MCAO. In total, 409 mRNAs were identified to be significantly upregulated and 255 were downregulated in ischemic NSCs compared with non-ischemic NSCs. Our study for the first time revealed differential lncRNA profiles and lncRNA–mRNA co-expression network in the NSCs that are associated with stroke-induced neurogenesis [80]. Furthermore, we demonstrated that the top upregulated lncRNA H19 mediates the proliferation and neuronal differentiation of NSCs by recruiting chromatin remodeling proteins and by regulating miR-675 expression to modulate neurogenesis-related transcription. H19 inactivation in NSCs significantly attenuates motor and cognitive function after stroke in vivo. In addition, we identified multiple lncRNAs that correlated with their adjacent genes such as Stat1 and GFAP, which are known regulators of NSCs [80]. However, mechanistic modulation of these lncRNAs in stroke-induced neurogenesis and neurological outcomes remains to be determined. Unveiling their mechanism will provide new insights into the epigenetic control of brain remodeling after cerebral ischemia.

### 3.7. Exosomal RNAs as Novel Regulators of the Adult Neurogenic Niche after Stroke

#### 3.7.1. Crosstalk between NSCs and Other Cell Types

Exosomes are endosomal origin membranous nanovesicles with a size of ~30–150 nm in diameter and are released by all living cells [126]. Exosomes mediate intercellular communication including neuron-glia and neuron-endothelial cells in the brain, by transferring proteins, lipids and genomic materials including miRNAs between source and target cells [126].

Adult neurogenesis is tightly controlled by the neurogenic niche, which provides a structural and molecular cue for NSC proliferation and the differentiation and functional integration of new neurons. We and other groups demonstrated that NSCs form close associations with peri-infarct blood vessels in a region of active vascular remodeling, in which angiogenesis is causally linked to post-stroke neurogenesis [127,128]. Angiogenic factors including vascular endothelial growth factor (VEGF) and angiopoietin 2 were reported to regulate the coupling of angiogenesis and neurogenesis [129,130,131]. In addition to NPCs, neuroblasts and blood vessels, the neurogenic niche includes astrocytes, microglia, ependymal cells, immune cells, etc. [132,133,134]. Interestingly, as noted above, multiple miRNAs abundant in the NSCs are also expressed in the neighboring cells, such as miR-146a and miR-124, which are co-expressed in NSCs and microglia [96,99]. However, the detailed molecular mechanism underlying their coupling after stroke remains unclear. Further investigation of the function of exosomes and RNA cargos in the communication of NSCs and adjacent cells after stroke may provide new therapeutic targets.

#### 3.7.2. Exosomal RNAs as a Potential Therapy in the Stroke

Compared to cell therapy, exosome administration has advantages, such as the virtually zero probability for developing a tumor or malignant transformation. Neural stem cells-derived exosomes via their RNA cargoes hold great potential to promote brain repair and functional recovery for stroke and other neurological injuries. Our group uncovered that treatment of stroke with mesenchymal stem cells (MSCs)-derived exosomes alone or tailored MSC-derived exosomes enriched with the miR17-92 cluster markedly increases neural plasticity and functional recovery after stroke via targeting PTEN to activate the PI3K/protein kinase B/mechanistic target of rapamycin/glycogen synthase kinase 3β signaling pathway in rats [135]. In line with our study, Yang et al. reported that systemic administration of rabies virus glycoprotein (RVG) modified MSC-exosomes fused to exosomal protein lysosome-associated membrane glycoprotein 2b (Lamp2b) and loaded with miR-124 protects against ischemic injury by inducing robust cortical neurogenesis [136]. In addition, exosomes from human urine-derived stem cells have been shown to enhance neurogenesis via the miR-26a/HDAC6 axis using an in vitro oxygen-glucose deprivation/reoxygenation (OGD/R) and an in vivo rat MCAO model [137]. MiR-124-loaded nanoparticles increase survival and neuronal differentiation of NSCs in vitro [138]. Exosomal lncRNAs also play an important role in the intercellular communication [139]. However, the mechanisms by which lncRNAs in exosome-mediated NSC communicate with other cells after stroke remain to be determined.

## 4. Conclusions and Future Perspective

**Figure 1:**
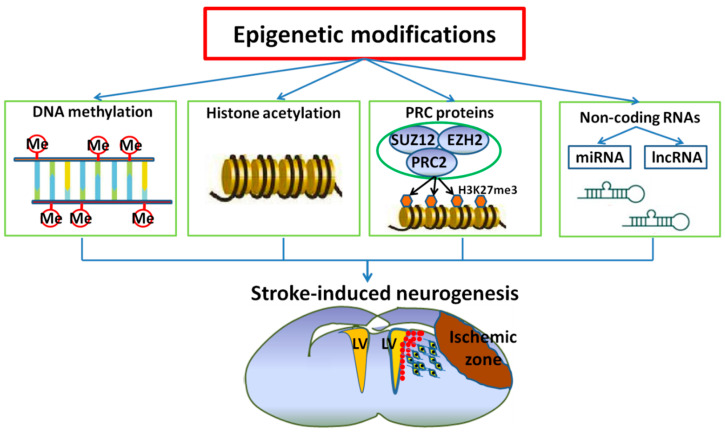
Proposed mechanistic models of the role of epigenetic changes including DNA methylation, histone acetylation, PCR core proteins, non-coding miRNAs and lncRNAs in the regulation of adult neurogenesis following stroke.

Epigenetic mechanisms have established roles in adult neurogenesis within the normal brain. These roles have been demonstrated via studies of neurodevelopment, learning and memory. In comparison, the role of such mechanisms in neuroplasticity following injury has not been clearly defined. Epigenetic changes have been investigated following stroke, but mostly in the context of injury evolution. In this brief overview, we seek to capture the emerging excitement from the increase of a new understanding of epigenetic molecular mechanisms and their relevance to NSC function and dysfunction (Figure 1). These roles need to be examined independently in the context of the injured brain to provide a comprehensive understanding of how repair processes contribute to late recovery and to identify plausible candidates important for the induction of stroke-induced plasticity. We consider this review to be a snapshot of where things stand at this point in time, with emerging new epigenetic mechanisms and targets. Further investigation of DNA methylation and demethylation, histone modifications and non-coding RNA regulation following stroke will provide important insights into the basic mechanisms of such plasticity and further enhance our understanding of the brain’s inherent regenerative capacity.

## Figures and Tables

**Table 1:** MiRNAs and the regulation of neurogenesis after stroke.

miRNAs	Target Genes/Regulated Pathways	Function	Citation
miR-124a	JAG1-Notch pathway	Reduce NPC proliferation and promote neuronal differentiation.	[96]
miR-17-92	PTEN, Enigma homolog 1 (ENH1)	Increase NPC proliferation and inhibit cell death.	[97]
miR-146a	IRAK1	Increase NPC proliferation and inhibit NPC death.	[99]
miR-126	PTPN/AKT-ERK pathway	Promote neurogenesis and improved neurobehavioral outcomes.	[100]
miR-195	Sema3A/Cdc42/JNK, Sema3A, NF-kB pathway	Anti-apoptosis, anti-inflammation, neurovascular Protection and promoting NSC proliferation and migration.	[101]
miR-9	HDAC4	Promote neuroprotective and regenerative efficacy.	[102]
miR-210	BDNF, COX10, ISCU/AMPK pathway, SOCS1-STAT3-VEGF-C Pathway	Affect proliferation, differentiation, apoptosis, mitochondrial function of NSCs, neurovacularization.	[103,104,105]
miR-365	Pax6	Inhibit neurogenesis.	[106]
miR-148b	wnt/β-catenin signaling	Attenuate proliferation and differentiation of NSCs.	[107]
miR-128-3p	Nrf2	Inhibit NSC proliferation and enhancement of oxidative stress.	[108]
miR-140-5p	IL1rap, IL1rapl1, VEGF and MEGF10	Inhibit neurogenesis and capillary density.	[109]
miR-27b	AMPK	Inhibit neurogenesis.	[110]
